# Supplementary material for: Are carbon emissions trading and green financial instruments synergistic? -Comprehensive quantitative research based on content analysis
Source: PLoS One. 2024 Mar 7;19(3):e0298601. doi: 10.1371/journal.pone.0298601 (PMC10919601; doi:10.1371/journal.pone.0298601)
Supplement: S1 Data — (ZIP) [file pone.0298601.s001.zip › Data/地级市Z_F工作报告.zip!/╔╜╬≈/╤⌠╚¬╩╨/└┤╫╘╣1⁄2╓┌║┼í╛┬φ┐╦╩2╛▌═°í┐_2016.docx]

各位代表：

　　现在，我代表市人民政府向大会报告工作，请予审议，并请市政协委员和其他列席人员提出意见。

　　一、“十二五”时期经济社会发展回顾

　　“十二五”时期是我市发展史上极不寻常的五年。五年来，面对严峻复杂的宏观经济环境，面对长期形成的深层次矛盾，面对艰巨繁重的改革发展稳定任务，全市上下深入贯彻落实党的十八大和十八届三中、四中、五中全会和习近平总书记系列重要讲话精神，按照省委“五句话”总要求和“六大发展”的战略部署，牢牢把握稳中求进工作总基调，着力稳增长、促改革、调结构、惠民生、防风险、保安全，攻坚克难，奋力拼搏，经济社会发展在克服困难中取得了新的成绩，基本完成了“十二五”确定的各项目标任务。

　　——我们千方百计稳增长，综合实力再上新台阶。过去的五年，特别是2013年以来，面对持续加大的经济下行压力，建立了部门、企业联席会议制度，加强对经济运行的分析调度。认真贯彻落实国家、省关于稳增长的各项政策措施，出台了金融支持实体经济发展意见等措施，设立了综合产业扶持基金、企业资金链应急保障资金和中小企业发展基金，重点稳定工业运行、支持煤炭脱困、鼓励中小微企业发展。激发有效需求，释放发展红利，挖掘增长潜能，多措并举，顶住下行压力，经济保持平稳运行。“十二五”末，全市地区生产总值达到595.7亿元，年均增长6.7%；全社会固定资产投资达到600.7亿元，年均增长18%；社会消费品零售总额达到288.3亿元，年均增长12.1%；公共财政预算收入达到44.2亿元，年均增长3.7%；城镇、农村常住居民人均可支配收入达到26414元、11494元，年均分别增长9.1%、11.9%。

　　——我们积极扩大投资需求，重点工程建设力度空前。“六位一体”推进项目建设，下大力实施了一批事关全局的重大基础设施和民生工程，投资对经济增长的贡献率达60%以上。开工建设总投资45.9亿元、总长79公里的阳大铁路，建成后将极大提升阳泉作为晋东区域中心城市的地位；启动实施总投资1.55亿元的城市饮用水水质改善工程，努力让群众喝上优质水、放心水；重启了总投资2.02亿元的娘子关二期供水工程，从根本上缓解全市水资源供需矛盾。大力开展招商引资，共签约外来投资项目867个，到位资金1404.9亿元，百度云计算、中广核风电等一批具有转型引领和辐射带动作用的大项目、好项目相继落户阳泉。大力发展“飞地经济”，全市“飞地”项目达到21个，总投资218亿元，天元废旧家电拆解、桃林沟欢乐世界等项目竣工投产。五年全市累计实施各类重点工程项目1115项，完成投资2259亿元。省级重点工程投资完成率连续四年进入全省前3名。

　　——我们统筹推进城乡发展，新型城镇化建设不断加快。加快推进旧城、新城、大县城、重点镇建设，城镇基础设施完成投资72.57亿元。大力实施道路畅通工程，泉西路、保晋路、洪城北路等建成通车，华盛桥、赛鱼桥等桥梁改造工程顺利完工。阳五、太阳、西环高速相继竣工通车，市域内形成了100公里的高速圈，高速公路密度达到6.27公里/100平方公里，位居全省第1。启动了生态新城建设，漾泉大道等一批路、水、气、暖工程正在加速推进。城市供水、燃气、集中供热普及率和生活垃圾无害化处理率分别达到100％、97.99％、95.98％和100%。启动并加快推进“智慧阳泉”建设，在全省率先实现主要公共场所无线互联网免费全覆盖。全市城镇化率达到65.86%，居全省第2。

　　——我们持续推动结构调整，资源型城市转型扎实有效。按照“稳定煤炭、加快电力、扩大煤化、做强装备、创新冶金、重组耐材、做大旅游”思路，积极推进传统产业改造提升和新兴产业发展壮大。加快重组整合矿井改造，建成现代化矿井5座。坚持变输煤为输煤、输电并重。五年来，建成、在建、获得“路条”及正在规划中的火电、风电、光伏发电项目总装机容量近800万千瓦，全部建成后总装机规模将达“千万千瓦级”。2×20万吨乙二醇项目一期即将竣工。兆丰铝业氧化铝二期70万吨、高精铝板带一期12.5万吨项目投产运行。大力发展煤矿电气、通用机械、特种石墨等装备制造业。积极发展高新技术产业，百度云计算一期3万台服务器正式投用。推进煤矸石、煤层气、粉煤灰和废旧家电的综合利用，全市资源综合利用企业达52家。加快发展旅游业，五年全市旅游总收入达611.9亿元，年均增长22.5%。三次产业比重由“十一五”末的1.5∶59.5∶39调整为1.7∶49.8∶48.5，结构进一步优化。

　　——我们加大强农惠农力度，“三农”工作成效明显。五年来，财政对“三农”投入累计达29.5亿元。积极实施产业富民工程，形成了以畜牧、核桃、蔬菜为主导，小杂粮、中药材、食用菌、红薯竞相发展的农业产业格局。“一村一品”专业村累计达321个。农民专业合作社、家庭农场分别发展到2271个、154家，土地流转面积11.3万亩。农产品加工销售收入达25亿元，是“十一五”末的3.4倍。产业扶贫和精准扶贫成效明显，全市农村脱贫人口达2.2万人。大力实施农村人居环境改善“四大工程”，完成县乡公路改造4395公里、农村街巷硬化6626公里；新建饮水安全工程303处，自来水入户率达95%；为611个村安装路灯12213盏；104个新农村建设重点推进村、3个集中连片示范区建设进展顺利，农村面貌焕然一新。

　　——我们大力发展社会事业，民生改善走在全省前列。在财政十分困难的情况下，民生支出占一般公共预算支出的比重由2010年的74.7%提高到2015年的82%，五年共兴办民生实事35件，实施了“七大民生工程”。创业就业促进工程。五年有2800多个行政、事业岗位面向社会公开招录。累计新增城镇就业12.02万人，转移农村劳动力5万人。城镇登记失业率控制在4%以内。社保提标扩面工程。在全省率先实现60岁以上农民基础养老金发放全覆盖。城乡居民基本医疗保险人均财政补助标准提高到380元。城镇社会保险参保率提高到97.83%，城乡低保、农村五保实现应保尽保，保障水平持续提高。城乡住房保障工程。累计开工新建各类保障房65219套，基本建成53181套，完成农村危房改造任务7030户。教育均衡提升工程。新（改、扩）建公办标准化幼儿园60所，改造农村幼儿园52所。在全省率先实现国家义务教育发展基本均衡县（区）全覆盖。职业技术学院新校区开工建设。太原理工大学阳泉学院成功“升本”，更名为“山西工程技术学院”。卫生强基固本工程。全市7所县级公立医院全部实行药品零差率销售。59所政府办基层医疗卫生机构、906个村卫生室和13个非政府办基层医疗卫生机构全部实施基本药物制度。城乡居民大病保险全省试点工作成效明显。文化惠民利民工程。覆盖城乡的现代公共文化服务体系基本建成。“文化惠民在山城”系列活动扎实开展，“一年一场戏”公益演出惠及130个偏远贫困村。我市作家刘慈欣作品《三体》获世界科幻大会雨果奖，这是亚洲作家首次获此殊荣。成功举办全国蹦床赛，我市体育健儿在国际、国内重大赛事上摘金夺银。平安阳泉创建工程。加强和创新社会治理，矿区社会服务管理经验在全国推广。积极推进“天网”工程建设，构建立体化治安防控网络。健全了信访工作机制和应急管理体系，信访形势明显好转，社会总体保持和谐稳定，人民群众的安全感和满意度进一步提升。毫不放松抓好安全生产，各类生产安全事故总死亡人数由“十一五”末的91人减少到60人，下降34%，首次杜绝了重特大事故。

　　——我们全面深化各领域改革，发展的活力动力进一步增强。抢抓转型综改试验区建设和获批“国家中小城市综合改革试点市”机遇，大力推进各领域改革创新。深化行政审批制度改革。全面推行“两集中、两到位”，七大类53项民生类服务项目办理时限压缩51.5%。公布运行了政府部门权责清单。扎实推进商事制度改革，除国家规定保留事项外，全部实现“先照后证”；全面推行“三证合一”、“一照一码”。深化国有企业改革。阳煤集团正式控股南煤集团。启动了市阀门厂、水泵厂“退城进园、搬迁改制”工作。市属国有照相馆、理发馆等服务企业全部完成民营化改制。深化地方金融改革。盂县农信社成功改制为农商行。全省首家专门服务“三农”的盂县汇民村镇银行挂牌成立。城商行改革迈出新步伐。上海股交中心阳泉企业挂牌孵化基地成立，挂牌企业达11家。深入实施创新驱动战略。在全省首家成立了科技银行，设立了3000万元的首支地市级高新技术投资发展基金。建成或在建科技孵化器5家，市科技孵化器成功申报为国家级科技企业孵化器，入孵企业累计达104家。我市成为国家级知识产权示范创建市，被列为全省首家创新型城市试点市。积极争取上级支持。先后争取到国家老工业区搬迁改造、独立工矿区改造搬迁资金4.66亿元、专项建设基金11.7亿元。新城建设项目获得国开行、农发行贷款额度27.3亿元，到位11亿元。

　　——我们狠抓节能减排和环境保护，生态文明建设屡创佳绩。五年组织实施重点节能项目94个，年节约52.2万吨标准煤，淘汰落后产能任务提前完成。大力实施大气污染防治净空工程，“十二五”六项减排指标任务全部完成。持续推进生态环境治理修复，完成矸山治理恢复3750亩、电厂脱硫除尘升级改造200.5万千瓦、脱硝治理133万千瓦。集中开展“土小”企业专项整治，1150座燃煤矾石竖窑、石灰窑全部依法取缔。新建市第二污水处理厂，完成4座污水处理厂提标改造，全市生活污水处理能力达到21.3万吨。五年完成各类营造林65.7万亩，森林蓄积量达到455.9万立方米，建成区绿化覆盖率达到41.4％。先后获得“国家园林城市”、“全国绿化模范城市”等荣誉称号。

　　——我们不断强化民主法治和自身建设，政府执行力和公信力显著提升。严格执行人大及其常委会的决议、决定，积极支持人民政协履行职能，共办理人大代表议案和建议905件、政协提案1710件。扎实推进“法治阳泉”建设，普法工作成效明显。不断完善基层民主制度，有序推进村委会换届选举。全力推进“六权治本”，制定了人事调动、预算外资金分配、工程项目招投标管理办法，全面公开部门预决算和“三公经费”信息，强化行政监察和审计监督。全面完成市县两级政府机构改革。公车改革扎实推进。深入开展党的群众路线教育实践活动、学习讨论落实活动和“三严三实”专题教育，着力构建作风建设长效机制。严格执行中央“八项规定”和国务院“约法三章”，坚决反对“四风”，“三公”经费支出由2012年的22468万元，压减至2015年的8407万元，年均降幅达27.94%。

　　与此同时，着力加强国防动员和后备力量建设，深入开展双拥工作，大力发展妇女儿童、老龄、残疾人和红十字会等事业，民族宗教工作受到国家表彰，外事、侨务、气象、地震、人防、档案、史志等工作取得新进展。

　　各位代表，“十二五”时期是我市改革开放以来发展极为曲折的时期之一，我们风雨同舟、众志成城，攻坚克难、砥砺奋进，走过了不平凡的历程。五年来，尽管经济增速有所放缓，一些指标完成不尽如人意，但民生改善没有削弱反而加强，教育、科技、卫生、体育等优势领域工作继续走在全省前列，人均收入、社会保障等继续保持先进水平，多年期盼的铁路、道路、供水、饮水、生态、环保等打基础、利长远的工程项目建成或正在推进。五年的成绩来之不易，经验弥足珍贵。要实现阳泉经济社会持续健康发展，必须坚持以经济建设为中心不动摇，始终把发展作为解决一切问题的关键，将贯彻落实中央、省精神与阳泉实际相结合，不断创新发展思路，完善发展举措，推动科学发展。必须坚持转型升级主线不偏离，以调结构、转方式为主线，不断改造提升传统产业，培育发展新兴产业，构建充满活力的多元产业支撑体系。必须坚持保障改善民生不懈怠，把人民对美好生活的向往作为我们的奋斗目标，多做打基础、利长远的实事好事，使发展成果更多更公平地惠及全市人民。必须坚持主动适应新常态不彷徨，既努力解决当前工作面临的新矛盾、新问题，又科学谋划事关长远发展的新路径、新办法，积蓄发展新动能，开创发展新境界。必须坚持深化改革创新不停步，以综改试验区建设为统领，抢抓各种政策机遇，持续不断推进重点领域改革创新，为推动发展提供强大的引擎。必须坚持全面从严治党、从严治吏不手软，认真履行“主体责任”，深入推进“六权治本”，坚决把纪律和规矩挺在前面，努力营造干事创业、风清气正的发展环境，不断开创我市经济社会发展新局面。

　　各位代表，五年来，能够取得这样的成绩实属不易。这是省委、省政府坚强领导、亲切关怀的结果，是市委统揽全局、科学决策的结果，是市人大、市政协大力支持、有效监督的结果，是全市干部群众团结协作、共同奋斗的结果。在此，我代表市人民政府，向全市人民，向驻地部队指战员、武警官兵、公安民警和中央、省驻地单位，向各民主党派、工商联、无党派人士、人民团体和所有关心支持阳泉改革发展的各界人士，表示崇高的敬意和衷心的感谢!

　　在肯定成绩的同时，我们也清醒地看到，我市经济社会发展还存在一些困难和问题，主要是：经济总量不大，发展不快，新的经济增长点不多；“一煤独大”、“一企独大”结构性矛盾仍然非常突出，传统产业产能过剩，接续产业发展不足，多元化支柱产业体系尚未形成；民营经济比重不大、活力不强、动力不足，大众创业的氛围尚未形成；园区建设滞后，制约项目建设的深层次问题还没有得到根本解决；城市基础设施欠账多，城市环境卫生问题还比较突出；政府职能转变仍显滞后，“四风”问题尚未根绝，廉政建设、法治建设需要进一步增强；一些干部担当意识不强，干事创业热情不足，工作中存在不敢作为、不会作为的现象。对此，我们将以高度的责任感和使命感，加倍努力，扎实工作，绝不辜负全市人民的期望和重托。

　　二、“十三五”时期经济社会发展的

　　指导思想和奋斗目标

　　 “十三五”时期，是全面建成小康社会的决胜阶段。当前，我国经济正在进行深度调整，我市作为典型的资源型地区和老工业基地，长期积累的深层次矛盾和问题日益凸显。但每一次危机都伴随着新的希望与重生，总体来讲，我们面临的仍然是一个机遇与挑战并存，机遇大于挑战的环境。我国经济长期向好的基本面没有改变，新常态下正在由原来快速发展向发展方式转变、由原来规模扩张向提高质量和效益转变。中央明确提出“五大发展”理念，并开出了推进供给侧结构性改革的经济治理良方，必将为经济发展开拓新空间、注入新活力。省委、省政府确立了实施“三个突破”、推动“六大发展”战略举措，持续净化政治生态，推进“六权治本”，为加快发展进一步理清了思路、营造了环境。我市区位优势明显、产业基础较好、城市化水平较高，产业结构调整和项目建设取得积极进展，先后争取到全国中小城市综合改革、国家老工业区搬迁改造和独立工矿区改造搬迁等一系列政策支持，发展的后发优势明显增强。综合判断，我市与全国全省一样，仍处于可以大有作为的重要战略机遇期。只要我们抓住机遇，迎难而上，就一定能够开创“十三五”发展的新局面。

　　根据市委十一届六次全会精神及“十三五”规划《建议》，市政府编制了“十三五”规划《纲要（草案）》，明确提出了“十三五”时期我市发展的指导思想，即：高举中国特色社会主义伟大旗帜，全面贯彻党的十八大和十八届三中、四中、五中全会精神，坚持以马克思列宁主义、毛泽东思想、邓小平理论、“三个代表”重要思想、科学发展观为指导，深入贯彻落实习近平总书记系列重要讲话精神，遵循“五位一体”总体布局和“四个全面”战略布局，坚持发展是第一要务和抓好党建是最大政绩，牢固树立并切实贯彻“五大发展”新理念，按照省委“五句话”总要求，主动适应经济发展新常态，以综改试验区建设为抓手，以转型升级为主线，以改革创新为动力，大力实施“三个突破”，着力推动创新发展、协调发展、绿色发展、开放发展、共享发展、廉洁和安全发展，确保全面建成小康社会，努力把我市建设成为山西向东开放桥头堡、晋东区域中心城、转型发展先行市、城乡统筹示范区。

　　今后五年全市经济社会发展的主要目标是：转型升级有新进展。经济结构调整与发展方式转变取得重大进展，服务业比重进一步加大，支柱产业多元化和大中小微企业协调发展格局初步形成。民营经济实力不断增强，科技支撑和创新驱动能力大幅提升，经济发展质量和效益明显提高。民生福祉有新增进。公共服务体系更加健全，社会保障体系更趋完善，基本公共服务均等化水平稳步提高，城乡居民收入持续增加。人民群众生活质量、健康水平进一步提高。文化软实力有新提升。文化事业整体水平、文化产业综合实力进一步提升，公共文化服务体系进一步完善。全民科学文化水平和精神文明素质普遍增强，城市文明程度明显提高，建成省级文明城市。生态建设有新成效。节能降耗、治污减排指标全面完成，生产方式和生活方式绿色低碳化水平显著提高。采煤沉陷区治理基本完成，森林覆盖率进一步提高，生态环境质量持续改善。城市面貌有新变化。“生态、智慧、宜业、宜居”的现代化城市建设取得明显成效。生态园林城市建设步伐加快，城市绿化覆盖率、人均公园绿地面积不断提高；互联网、物联网、云计算等信息技术与城市管理、市民生活高度融合，城市管理和运行实现智慧化；大众创业、万众创新蓬勃发展，都市经济规模不断扩大；城市基础设施和文化教育、休闲娱乐、养老医疗等大众化服务设施进一步完善。改革开放有新突破。重点领域和关键环节改革取得决定性成果，各项改革试点工作取得重大进展。对外开放的广度和深度不断拓展，积极参与京津冀、环渤海、长三角、珠三角乃至“一带一路”等更大范围的协作分工，并取得实效。依法治市有新局面。法制建设扎实推进。法治政府基本建成，司法体制改革基本到位，司法公信力明显提高。全民法治意识不断增强，社会治理体系和治理能力现代化水平不断提高，社会更加和谐稳定。

　　各位代表！如期实现全面建成小康社会目标，承载着全市人民对美好生活的殷切向往。“十三五”规划《纲要（草案）》已印发各位代表，经本次会议审议通过后，我们将全力抓好落实。我们坚信，在省委、省政府和市委的坚强领导下，通过全市人民的奋力拼搏、共同努力，一定能够夺取全面建成小康社会决胜阶段的伟大胜利！

　　三、2016年工作安排

　　2016年是全面建成小康社会决胜阶段的开局之年，也是推进结构性改革的攻坚之年。我们要全面贯彻党的十八届五中全会精神，按照中央经济工作会议及省委十届七次全会、全省经济工作会议要求，认真落实市委十一届六次全会、全市经济工作会部署，认识、适应和引领经济发展新常态，坚持稳中求进工作总基调，坚持稳增长、调结构、惠民生、防风险，落实宏观政策要稳、产业政策要准、微观政策要活、改革政策要实、社会政策要托底的要求，着力加强供给侧结构性改革，去产能、去库存、去杠杆、降成本、补短板，实施“三个突破”，推动“六大发展”，努力实现“十三五”经济社会发展良好开局。

　　综合考虑各方面因素，2016年我市经济社会发展的主要预期指标是：地区生产总值增长6%左右，全社会固定资产投资增长15%，规模以上工业增加值增长1.3%，社会消费品零售总额增长5.5%，城镇新增就业岗位1.9万个，城乡常住居民人均可支配收入增长6%左右和6%以上，公共预算收入降幅控制在9%之内，居民消费价格涨幅控制在3%左右，城镇登记失业率控制在4.2%以内。约束性指标：包括万元地区生产总值能耗、二氧化碳排放量、用水量、主要污染物减排、空气质量优良天数比例、劣V类水体比例、新增建设用地降幅、农村贫困人口脱贫人数、城市棚户区住房改造数量等，待省确定后，分解下达各县区执行。

　　为实现上述目标，重点要做好以下几方面工作：

　　（一）全力推动供给侧结构性改革。

　　把加快供给侧结构性改革作为推进经济转型升级的关键举措，坚持28字调产思路，突出化解过剩产能，不断培育和壮大新的经济增长点。

　　努力促进煤炭行业转型脱困。严格执行省“十三五”期间现有生产煤矿产能核定“只减不增”要求，严控新建煤矿新增能力，坚决化解煤炭过剩产能。通过提高环保、安全、技术标准，对落后产能坚决依法依规淘汰关闭。推进煤炭深加工，加大洗选、配送比例，提高煤炭附加值。推进煤炭资源综合利用，大力发展煤化工、煤建材、煤电铝，延伸产业链条，发掘新的经济增长点，提高煤炭就地转化率。积极争取国家、省对煤层气等煤炭共生伴生资源开发利用一体化的政策支持，大力实施煤矿瓦斯抽采全覆盖工程，提升煤矿瓦斯综合利用水平，年内完成平定阳胜煤业5兆瓦低浓度瓦斯发电和郊区保安煤业2500万立方瓦斯并网工程。提高煤矸石、煤泥和矿井水的综合利用水平。

　　加快推进传统非煤产业改造升级。要抓住国家推进电力体制改革政策机遇，积极推进火力发电和低热值煤发电，发展大容量、高参数超临界、超超临界燃煤发电机组，做大产业规模，加快阳煤远盛2×35万千瓦、西上庄2×66万千瓦和国际能源裕光煤电2×100万千瓦等项目建设。着手提高电力消纳能力，促进煤电铝、煤化工等现代高载能行业消化吸纳，加快盂县—河北500千伏外送电通道建设。要以煤矿机械、煤矿电气和通用机械为重点，年内完成盛旺管业25万吨球墨铸管土建及设备安装工程，积极推动天元家电生产和废旧家电拆解项目试生产。利用好大用户直供电和企业自备电政策，帮助扶持铝工业降低成本、提质增效，推进兆丰铝业20万吨高精铝板带二期项目开工。要大力推进耐火企业整合重组和转型升级，着力开发中高档耐火材料和中低密度陶粒砂产品，加快金土耐材前期工作，提高产业集中度和市场竞争力。

　　积极培育发展新兴产业。大力推进太阳能、煤层气、风能等新能源的开发利用。加快采煤沉陷区国家先进技术光伏发电示范基地项目前期准备工作，年内力争开工建设一期总规模105万千瓦的11个基地项目。积极推进中广核盂县风电三期2万千瓦项目，确保年内并网发电。加快粉末冶金、磁性材料、陶瓷等新材料产业发展，稳定贝特瑞金刚石纯化粉现有生产规模，加快山西力腾高科铈磁体生产及技术升级项目建设。以高效节能电机、节能阀门、LED照明系统等为重点，促进节能环保产业快速成长，推进中电龙华热泵回收供暖示范等项目建设。依托百度云计算项目，积极引进、发展与之相关的上、下游产业和产品，逐步形成从硬件、软件到信息技术集成服务的完整产业链。

　　加快发展现代服务业。落实中央、省促进现代服务业发展的各项政策措施，统筹推进生产性服务业和生活性服务业全面发展。发挥区位优势，大力发展现代物流业，加快推进晋东商贸物流园、蒙牛物流基地等项目建设。大力发展文化旅游产业，支持刻花瓷、煤雕、砂器等本土特色文化产业做大做强，促进文化与旅游融合发展。打造精品旅游线路，不断提升藏山、娘子关、桃林沟等景区知名度和影响力，加快实施大汖温泉度假景区二期、红岩岭自然风景区等项目建设，年内完成旅游配套设施改造，正式对外开放。提升健康养老服务水平，推动医疗卫生和养老服务融合发展。积极扩大消费需求，以农村乡镇和城市社区为重点，增强乡镇商贸配送中心辐射能力。巩固、发挥“15分钟便民商圈”功能，推动商业综合体、社区多业态店等新型业态发展，不断释放消费潜能。大力发展电子商务，培育电子商务经营主体，加快电子商务园区建设，依托阳泉特产网等平台，重点推动向日葵信息技术、乐村淘电子商务等电商企业发展。

　　全力保障企业稳定运行。认真贯彻落实中央、省工业企业降成本、增效益、稳运行的各项政策措施，建立健全企业减负机制。对重点企业、困难企业施行“一企一策”，分门别类采取对策，最大限度解决企业的实际困难和问题，帮助企业渡过难关。坚持与阳煤集团的联席会议制度，努力搞好协调服务，促进企业健康发展，实现企地共赢。引导企业强化管理、创新经营理念，进一步降低生产、交易、物流等成本，提高抗风险能力。鼓励企业开展立体化营销，努力化解销售难、回款难问题，促进产销平稳衔接。继续实施出口信保统保政策，帮助企业开拓国际市场，扩大外贸市场份额。

　　（二）大力推进重点项目建设。

　　以产业园区建设为突破口，奋力推进项目建设。今年全市固定资产投资任务为691亿元，重点抓好铁路、公路、低热值煤发电等“十大领域”197个项目，突出抓好阳大铁路、宁波北路等十大标志性工程。

　　积极推进园区建设。园区建设是我市经济发展的短板。要按照“政府主导、市场运作、招商为先、项目为重”的思路，高标准规划建设产业园区，吸引更多的资金、项目落户阳泉。平定、盂县、郊区要高标准规划建设一至两个园区，同时积极推动龙川工业园、鑫磊循环经济产业园等现有园区上档升级，全力提升“五通一平”建设水平。开发区要利用扩区机遇，加大东区工业园建设步伐。城、矿两区要用足用好“两区”建设政策机遇，积极推动市区工业企业退城入园，腾出发展空间，实现“退二进三”，努力提升中心城市的综合服务功能。

　　多渠道筹措项目资金。一要向上争取。用足用好上级政策，积极作为，主动对接，继续下大力争取国家专项建设基金、“两区”建设资金、中央预算内投资的支持。二要市场运作。采用PPP等模式，建立和完善为重点工程项目建设服务的信用担保体系，广泛引导社会资本投入到项目建设中。利用好多层次资本市场，加大企业直接融资力度。三要招商引资。借助我市区位优势，积极对接东部沿海发达地区，主动融入环渤海、京津冀以及石家庄、太原都市圈发展战略，承接产业转移，提供配套服务，实现梯次发展。实施精准招商、全产业链招商、集群化招商，强化以商招商、专业队招商，积极参加能博会、厦洽会、高交会等大型展会，发挥好委托代理机构作用，大力引进资金、项目、技术和人才。

　　完善项目推进机制。进一步减少前置审批事项，实行清单式管理，认真做好投资项目在线审批监管。强化“六位一体”工作考核机制，完善重点工程调度、投资运行分析等制度。继续实行月考核、月排名、月报告制度，通过排名、公示、约谈等办法，鼓励先进、鞭策后进。落实项目推进的主体责任，突出对固定资产投资、重点工程建设完成情况的目标责任考核，充分调动各县区、各部门以及项目单位工作积极性，确保项目建设稳步推进。

　　（三）不断提高新型城镇化水平。

　　加快完善规划体系。按照统筹城乡要求，做好新型城镇化顶层设计与相关规划的衔接，积极推进“多规合一”，重点加快《市区与平定同城化发展规划》、《海绵城市建设规划》、《老城区改造规划》的编制工作，不断完善各类城乡规划。强化规划执行，进一步完善联动执法机制，加强规划审批后的跟踪监督和执法检查，对违规审批的部门、单位和失职、渎职者严肃问责，对未批先建、违规修建者坚决查处，切实维护规划的权威性和严肃性。

　　加强基础设施建设。统筹推进旧城提质、新城开发。重点加快城市供排水、供热、供气等老旧管网改造，加强城市地下综合管廊、垃圾处理等工程建设，完善城市基本功能。抓好宁波北路、平阳街、洪城北路北延、漾泉大道一期、二期等工程建设，优化城市主干路网。推动阳大铁路、城市综合交通枢纽、太旧高速阳泉南连接线工程等项目实施，提高城市的通达性。加快晋东化工厂、蔬菜批发市场等搬迁项目进度，实现城市片区功能再造。同时，继续推进大县城和重点镇建设，不断完善城镇基础设施和社会服务功能。

　　大力推进城中村、棚户区改造。学习借鉴太原先进经验，统筹协调资金、土地等要素，建立融资平台，做深做细群众工作，扎实推进今年确定的驼岭头、老虎沟、南窑庄等11个城中村改造，力争年内实现大的突破，坚决杜绝现代城市中的破烂不堪现象。同时，抓住国家政策性机遇，加快推进简子沟、余积粮沟等棚户区改造项目，让困难群众早日搬进新居。

　　广泛开展环境综合整治。城市要大力开展雨污分流改造、污水配套管网、垃圾处置设施建设等工作。加大市容市貌、环境卫生、交通秩序“三项”整治力度，做好“三线”入地工作。重点打造南大街、义平路2条样板街路，形成有特色、有品位的城市街路。推进综合执法，下移执法重心，实现城市管理执法工作全覆盖。按照国家生态园林城市创建标准，有空造绿、见缝插绿，构筑绿化精品，力争建一处绿一处，建一处美一处。加快智慧阳泉建设，按照“创新、实用、共享、便捷”原则，推动智慧公交、智慧医疗、智慧食药监管建设，启动数字城管，从根本上提升城市管理档次。深化省级文明城市创建，进一步改善城市形象，力争早日跨入省级文明城市行列。农村要全面抓好“四好农村路”建设，重点推进农村公路安全生命防护工程和村通水泥（油）路工程。积极实施好3个县（区）、10个乡镇、15个村的采煤沉陷区治理和地质灾害避让搬迁工作，完成农村危房改造1600户。继续抓好乡村清洁工作，实施好14个村的乡村污水治理工程，创建乡村清洁达标村270个。推进美丽宜居示范村“三级联创”工作，力争再有3个村进入省级美丽宜居示范村行列。

　　下大力消化房地产库存。落实国家、省购房补贴政策，鼓励城镇居民改善住房条件，支持农业转移人口在城镇购房。推进住房体制改革，建立购租并举的住房制度，加大棚户区改造货币化安置力度。打通商品房和公租房供需通道，扩大公租房保障范围。通过系列政策引导，稳定房地产市场，确保完成省定销售面积增幅不低于20%的去库存任务。

　　各位代表，城市发展带动整个经济社会发展，城市建设已成为现代化建设的重要引擎，城市在经济社会发展、民生改善中发挥着越来越重要的作用。当前，我市城市建设和管理任务十分艰巨，但我们改变城市面貌、创造良好生活环境的决心更大！我们将举全市之力，充分调动各方面资源和力量，努力把阳泉建设成为生态、智慧、宜业、宜居的现代化城市！

　　（四）高度重视做好“三农”和脱贫攻坚工作。

　　夯实农业发展基础。积极开展中低产田改造，完成高标准农田建设3万亩、膜下滴灌4600亩，确保粮食产量稳定在2.1亿公斤以上。加大抗旱应急水源建设力度，年内完成平定岭南河、岔口、后底沟、原坪等水库除险加固工程，开工建设盂县乌河水库、檀山沟水库。加强农技队伍建设。同时，继续深化农村改革。全面完成土地承包经营权确权登记颁证工作，推动土地经营权规范有序流转。积极推进农村集体产权制度改革和经营体制创新，稳妥开展农村承包土地经营权和农民住房财产权抵押贷款试点。做好第三次全国农业普查工作。

　　大力发展特色现代农业。保持设施蔬菜种植面积在1.4万亩以上。发挥康泰来公司品牌效应，大力发展食用菌，不断扩大市场份额。促进核桃产业提质增效，年内完成高接换优5000亩。积极发展水果、中药材、蜂业等特色产业。小杂粮总产量达到4万吨以上。加快发展猪鸡牛羊等养殖业，肉蛋奶产量稳步提升。巩固发展50个“一村一品”专业村。支持裕盛源醋业、三来食品等龙头企业发展，全市农产品加工企业年销售收入增加15%以上。积极创建国家和省级农业示范园，促进粮经饲统筹、种养加结合、一二三产融合，抓好汉能集团胡家庄光伏基地等重点项目建设。大力发展休闲度假、旅游观光、养生养老、农耕体验、乡村手工艺等潜力产业。

　　坚决打赢脱贫攻坚硬仗。大力实施精准扶贫，对贫困人口进行甄别，摸清底数，精准定位扶持对象。健全完善市县抓落实的领导机制，建立贫困村互助合作基金，整合涉农资金，加大扶贫资金支持力度。更加注重“造血”扶贫，根据实际情况有针对性地实施产业扶持、技能扶持。重视发挥广大基层干部群众的首创精神，鼓励贫困群众树立自力更生观念，真正实现可持续发展。今年完成50个村、1万人的脱贫任务，其中产业扶贫脱贫7千人、易地搬迁脱贫1千人、教育扶贫脱贫1千人、社会保障兜底1千人。

　　各位代表，脱贫攻坚、全面小康是党中央交给我们的重大历史任务，是“十三五”的头等大事和第一民生工程。我们必须以更大的决心、更明确的思路、更精准的举措，坚决打赢脱贫攻坚战，确保到2018年，全市农村贫困人口在全省率先整体脱贫，决不让一户落伍、一人掉队。

　　（五）全面深化改革创新。

　　做大做强民营经济。着力在创优环境上出实招，认真贯彻落实省、市加快民营经济发展的政策措施，坚持领导干部联系民营企业机制，扎实开展民营经济“待批项目大起底”活动，认真解决企业发展中的困难和问题。着力在扩大规模上下功夫，继续大力实施中小微企业成长工程，筛选一批成长性好、竞争力强的民营企业进行重点扶持，集中培育一批“小升规”企业；积极推广平定金潭小微企业孵化基地经验和做法，规划建设一批基础设施完善的中小微企业园区和孵化基地。着力在增强活力上见成效，鼓励企业开展产学研联合创新，积极发展众创、众包、众扶、众筹空间，支持民间资本投资教育、医疗、养老等社会领域。通过扎实有效的工作，不断提升民营经济在经济总量中的比重，使之真正成为我市推动转型的中坚力量、促进升级的动力源泉。

　　加快推动科技创新。加快完善全市科技孵化体系，重点推进市高新技术创业园孵化基地扩容提质，支持县区和企业的孵化器建设。培育和发展高新技术企业，鼓励规上企业与国家级科研院所进行深度合作。在煤层气抽采技术、煤层气综合利用、煤机装备制造、石油支撑剂等领域加快实施一批重大科技专项和重点科技项目，争取突破一些关键技术。运用好高新技术产业股权投资基金，调动银行、社会资金投向科技型企业。加强创新人才队伍建设，利用好海外博士阳泉行活动平台，引老乡、回故乡、建家乡。

　　全力推进金融振兴。尽快完成城市商业银行增资扩股后续审批工作，加快推进平定、郊区农信社改制。积极推动企业上市，年内完成云泉岩土工程、天元绿环科技2户企业新三板挂牌，力争每个县区都要有一个新三板上市企业。完善融资担保体系，做大做强现有政策性融资担保公司，下大力破解中小企业融资难、融资贵的问题。做好金融机构引进工作，推动晋商银行阳泉分行、平定昌都村镇银行早日正式营业。着力发展债券等金融产品市场，引导和支持企业充分运用私募债、短期融资债等方式融资。加强和改进金融监管，确保地方金融稳定。

　　深化国资国企改革。水泵、阀门等重点企业“退城进园、搬迁改制”工作今年要迈出新步伐。长期停产、复活无望的“僵尸”企业，要采取多种方式，逐步退市。加快市直党政机关与所办企业脱钩改制工作，推进市属经营性国有资产统一监管。抓好企业财务等重大信息公开工作。加强对企业资产租赁管理、产权交易监管，确保国有资产保值增值。积极推进市属国有企业负责人薪酬制度改革。

　　同时，统筹推进转型综改工作，实施好2016年“9911”行动计划。积极推进财税体制、污染者付费制度改革，不断提升发展的新动力。

　　（六）强化节能减排和生态环保。

　　深化大气污染防治。巩固“土小”企业专项整治成果，对相关企业及责任人建立档案，明确责任，终身追责，决不允许死灰复燃。全面强化建筑施工工地、公路等重点领域扬尘监管，对不达要求的工地坚决停工整治，对公路管理不到位、监督不力的责任人严肃问责。加大矸山治理力度，按照“谁开采谁治理、谁生产谁负责”的原则，督促相关企业进行彻底整治，做到不欠新帐、要还旧账。不断强化环保执法，严厉打击环保设备闲置、超标排放等违法行为，严格落实按日计罚、查封扣押、限产限排等规定要求，打出最严环保执法组合拳。

　　加强生态建设。大力实施重点造林工程，全年完成各类营造林12万亩。扎实推进水保生态建设，完成水土流失治理面积45.8平方公里。建设市污水处理厂污泥处置中心。严格落实水源地保护措施，加快娘子关饮用水水源地污染企业搬迁。大力推进“五水同治”，优先保护饮用水，突出治理流域水，监督考核跨界水，积极防治地下水，全面处理污废水。

　　加大节能减排力度。大力推进清洁生产，坚决抑制高耗能、高污染产业增长，推动工业企业走上低碳绿色发展道路，确保完成省定各项约束性指标任务。积极推进工业能效提升工程,推行合同能源管理，促使企业节能降耗。继续推动煤矸石、粉煤灰等大宗工业固体废弃物和焦炉煤气等的资源化利用。支持企业实施锅炉窑炉改造、电机系统节能、能量系统优化、余热余压利用等节能改造工程。

　　（七）毫不放松抓好安全生产和社会稳定工作。

　　完善安全监管责任体系建设。按照“党政同责、一岗双责、失职追责”要求，进一步厘清各部门安全监管职责，做到管行业必须管安全、管业务必须管安全、管生产经营必须管安全。推动企业主体责任落实，督促指导企业做到安全责任、安全投入、安全培训、安全管理、应急救援“五到位”。严格落实目标责任考核“一票否决制”，切实执行安全生产不良记录“黑名单”制度，严查事故，严肃问责。

　　深化安全生产专项整治。继续坚持“456”工作法，加强隐患排查治理，经常性开展“四不两直”安全检查，推动多部门联合整治、联合除患、联合执法。落实企业安全生产ABC分类分级管理制度、挂牌督办制度，持续强化煤矿、道路交通、非煤矿山、危险化学品、建筑施工、油气输送管道、特种设备、学校、旅游、消防等重点行业和领域的安全监管，继续保持打击私挖滥采的高压态势，坚决杜绝重特大事故，减少一般性事故，防止意外事故。

　　加强和创新社会治理。深入开展“平安阳泉”建设，继续实施“天网”工程，加强社会治安综合治理。依法有序抓好信访维稳工作，及时妥善处理群众诉求。健全完善应急管理体系，进一步提升突发事件处置能力和水平。始终保持高压态势，深入开展“打黑除恶”斗争。推广矿区加强和创新社会治理工作经验，大力支持城区开展“全国社区治理和服务创新实验区”建设工作，建立专业化社区工作者队伍，推动服务和管理力量向基层倾斜。强化市场监管，狠抓食品药品综合治理。

　　（八）着力保障和改善民生。

　　促进各类教育协调发展。扩大学前教育资源，新改扩建10所农村幼儿园。巩固义务教育均衡发展成果。继续改善普通高中办学条件，推进教育教学改革。加快阳泉职业技术学院新校区建设，着力构建现代职业教育体系。认真落实特殊教育提升计划。进一步改善和优化教师队伍结构，全面提高教育教学质量。

　　提升公共文化服务水平。深化公益性文化事业单位内部改革，发展新闻出版、广播影视、文学艺术事业。开展好直播卫星“户户通”、农村公益电影放映等文化惠民工程。持续推动基层文化设施提档升级，加大市级“五馆一院”、县级“三馆一院”建设力度。加强网上思想文化阵地建设，净化网络环境。深入开展全民健身系列活动，不断完善竞技体育运动训练“三级网络”体系。

　　大力发展医疗卫生事业。不断深化医药卫生体制改革，巩固县级公立医院综合改革成果，推进市级公立医院综合改革试点工作，引深基层医疗卫生机构综合改革。继续提高基本公共卫生服务项目财政补助标准。新农合参合率保持在99%以上，完善城乡居民大病保险制度。推进优质医疗资源下沉，创新医疗联合体建设运营模式，全面实施分级诊疗。健全卫生计生综合监督体系，规范医疗卫生服务行为。巩固完善基本药物制度，推进公立医院药品阳光采购工作。

　　着力稳定和扩大就业。落实就业优先战略，深化创业型城市创建活动，完善创业扶持政策。继续实行政府购买基层公共服务岗位、招聘农村特岗教师、就业见习等措施，吸纳高校毕业生就业。做好农村转移劳动力、城镇失业人员、退役军人等群体就业工作，托底帮扶就业困难人员。加强就业培训，推行终身职业技能培训制度，实施职业培训全覆盖计划。

　　完善社会保障体系。稳步推进机关事业单位、企业基本养老保险制度并轨运行，完善相关配套政策，逐步提高退休人员基本养老金待遇水平。适当提高大病保险人均筹资水平和报销比例。完善重特大疾病医疗救助政策。落实经济困难高龄与失能老年人补贴以及百岁以上老年人补贴新标准。全面实施困难残疾人生活补贴和重度残疾人护理补贴制度。继续提高城乡低保标准，推动农村低保标准与国家扶贫标准相衔接。

　　努力增加城乡居民收入。依法推进企业工资集体协商，发布企业工资指导线，适时适度调整全市最低工资标准。健全农民工工资支付保障机制。调整机关事业单位基本工资标准，落实县以下机关公务员职务职级并行制度和乡镇工作补贴。通过加快农村产权改革、提高农民社保标准和政策补贴、发放农民冬季取暖补贴等，多渠道增加农民收入。

　　支持国防和军队建设，加强国防动员和后备力量建设，做好双拥、优抚安置和人防工作，推进军民融合深度发展。发展妇女儿童、老龄、残疾人和红十字会等事业。做好地震、民族宗教、外事、侨务、档案、史志等工作。

　　各位代表，越是经济下行，我们越要坚持共享发展，越要高度关注民生，把民生改善放在心上、抓在手中，让人民群众有更多的获得感和幸福感！

　　（九）切实加强政府自身建设。

　　加强党风廉政建设。按照全面从严治党要求，全面落实“主体责任”，认真履行“一岗双责”，深入推进政府系统党风廉政建设和反腐败斗争。坚持把纪律和规矩挺在前面，认真贯彻《廉洁自律准则》和《纪律处分条例》，严格执行廉洁自律各项规定，做到对党忠诚，廉洁自律。完善政府系统行政权力、防治腐败制约机制，强化重点部门和岗位廉政风险防控。严查群众身边的腐败问题，有腐必反、有贪必肃，全力营造风清气正、干事创业的良好氛围。

　　着力转变政府职能。继续深化行政审批制度改革，精简办事程序，缩短办事时限，加强事中事后监管，全面清理规范行政审批中介服务，努力形成权责一致、分工合理、决策科学、执行顺畅、监督有力的行政管理体制。加强电子政务建设，积极推进网上审批，让群众少跑路、少受累，让市场主体切实感受到方便和快捷。继续推进政务公开，加强软环境建设，全面提升服务水平。

　　严格做到依法行政。深入推进“六权治本”，坚持法定职责必须为、法无授权不可为。自觉接受人大及其常委会的监督和政协的民主监督，加强行政监察，强化审计监督，充分发挥舆论监督、群众监督作用。加强与各民主党派、工商联、无党派人士联系，积极支持工会、共青团、妇联等群众团体开展工作。强化依法决策、依法施政，不断提高政府工作法治化水平。

　　切实转变工作作风。巩固“三严三实”专题教育成果，扎实开展“两学一做”学习教育，坚持“双学”，深入落实中央八项规定和国务院“约法三章”，坚持不懈纠正“四风”，着力推进作风建设常态化、长效化。加大绩效考核和行政问责力度，严肃追究懒政怠政者责任，坚决扼制“想当官不想干事，要待遇不要作为”现象。注重工作时效性，发扬事在人为、积极进取的精神，“马上就办，真抓实干”，确保定一件、干一件，干一件、成一件。各级干部必须坚持以上率下，增强引领和推动发展的能力，更加注重汲取和借鉴基层群众创造的新鲜经验和丰富智慧，说实话、办实事、求实效，做到“头顶有戒、心中有数、眼里有活、手上有招、脚下有根”，以实实在在的工作成效取信于民。

　　当前，面对持续加大的经济下行压力，面对日益突出的财政收支矛盾，各级政府部门必须真正树立起过“紧日子”的思想，切实压缩开支，节俭办事，花好每一分钱，把有限的财力用在“保运转、保稳定、保重点、保民生”上。所有工作人员都要与全市人民同呼吸、共命运，上下同心，共克时艰，扎实走好“十三五”开局的第一步。

　　各位代表！阳泉山川秀美、文化厚重、人民勤劳、民风淳朴。无论在艰苦卓绝的革命战争年代，还是在激情燃烧的和平建设时期，全市上下不懈奋斗，取得了令人鼓舞的成就。我们坚信，有历届市委、市政府奠定的良好基础，有区位、资源和产业配套的比较优势，有广大干部群众团结拼搏的昂扬斗志，我们一定能够披荆斩棘，勇往直前，谱写出“十三五”发展的壮丽篇章！

　　各位代表，奋斗成就梦想，实干铸就辉煌。让我们更加紧密地团结在以习近平同志为总书记的党中央周围，在省委、省政府和市委的坚强领导下，坚定信心、迎难而上，以苦干创造实绩，以实干造福人民，不断开创经济社会发展新局面，为夺取全面建成小康社会决胜阶段新胜利而努力奋斗！
